# Amorphous calcium phosphate, the lack of order is an abundance of possibilities

**DOI:** 10.1016/j.bbiosy.2021.100037

**Published:** 2021-12-28

**Authors:** Lorenzo Degli Esposti, Michele Iafisco

**Affiliations:** Institute of Science and Technology for Ceramics, National Research Council, Via Granarolo 64, Faenza 48018, Italy

**Keywords:** ACP, Amorphous calcium phosphate, CaP, Calcium phosphate, CPP, Casein phosphopeptide, Amorphous calcium phosphates, Dental remineralization, Bone cements, Coatings, Drug delivery, Biomimetics

## Abstract

For almost three decades from its discovery, amorphous calcium phosphate (ACP) was not considered a suitable biomaterial due to its structural instability. Thanks to its unique properties in respect to crystalline calcium phosphate phases, nowadays ACP is used in promising devices for hard tissue regeneration. Here we have highlighted the features of ACP that were harnessed to create excellent biomaterials for dental remineralization, self-setting bone cements, drug delivery, and coatings of prostheses. Its current limitations as well as future perspectives of development were concisely described. Despite more research works are needed, we envisage that the future of ACP is bright.

## Highlights

• In the past, amorphous calcium phosphate (ACP) was not considered a usable material due to its metastability.
• The ability to crystallize in water and release ions have made ACP an excellent material for dental remineralization.
• ACP is a suitable biomaterial for coatings of prostheses and self-setting injectable bone cements.
• Current limitations of ACP are the need of stabilizing agents for long-term storage, and the difficulties to investigate its amorphous structure.
• The unique properties of ACP make it one of the most interesting materials to develop of advanced biomaterials.

## Introduction

Biomaterials were originally conceived to provide substances that can replace the damaged tissues. They were prepared to match as close as possible the physical, chemical, and mechanical properties of natural tissues. In the case of bones and teeth substitution, this research field prompted the detailed study of crystalline calcium phosphates (CaPs), mainly in the form of hydroxyapatite.

In 1965, studying the synthesis of hydroxyapatite, Aaron S. Posner discovered that as first precipitate a non-crystalline CaP product formed [1], but the relevance of this finding was appreciated some decades later. In fact, for several years after Posner's discovery amorphous calcium phosphate (ACP) was not considered a usable material due to is intrinsic instability (i.e., tendency to spontaneously evolve to a crystalline phase) and especially due to its different structure in comparison to biogenic crystalline CaPs. During the first decade of the twenty-first century, a paradigm shift in the development of biomaterials occurred, namely the activity of materials was reconsidered moving from the tissue substitution to regeneration. Therefore, the interest in how the human mineralized tissues form and biomaterials can induce tissue formation and cellular responses has resurrected the attention to ACP. Indeed in the same years, thanks to the improvement of the investigation tools at the nanoscale (i.e. high-resolution microscopies, synchrotron based investigation techniques, etc.) it was found that biogenic CaPs in vertebrates are formed through the crystallization of an ACP precursor. It is nowadays well recognized that CaP nanocrystals of bones and teeth are not formed through a classical ion-by-ion crystallization mechanism, but as first step ACP nanoparticles are deposited in the mineralization site followed by crystallization. Robinson et al. [2] were one of the first investigators to prove that in teeth spherical ACP nanoparticles self-assemble into chain-like macrostructures and then crystallize into hydroxyapatite to form enamel rod crystals. These findings were not only important to comprehend how the physiological and pathological minerals form but were also a source of inspiration to design new advanced biomaterials.

## State of the art

**Fig. 1:**
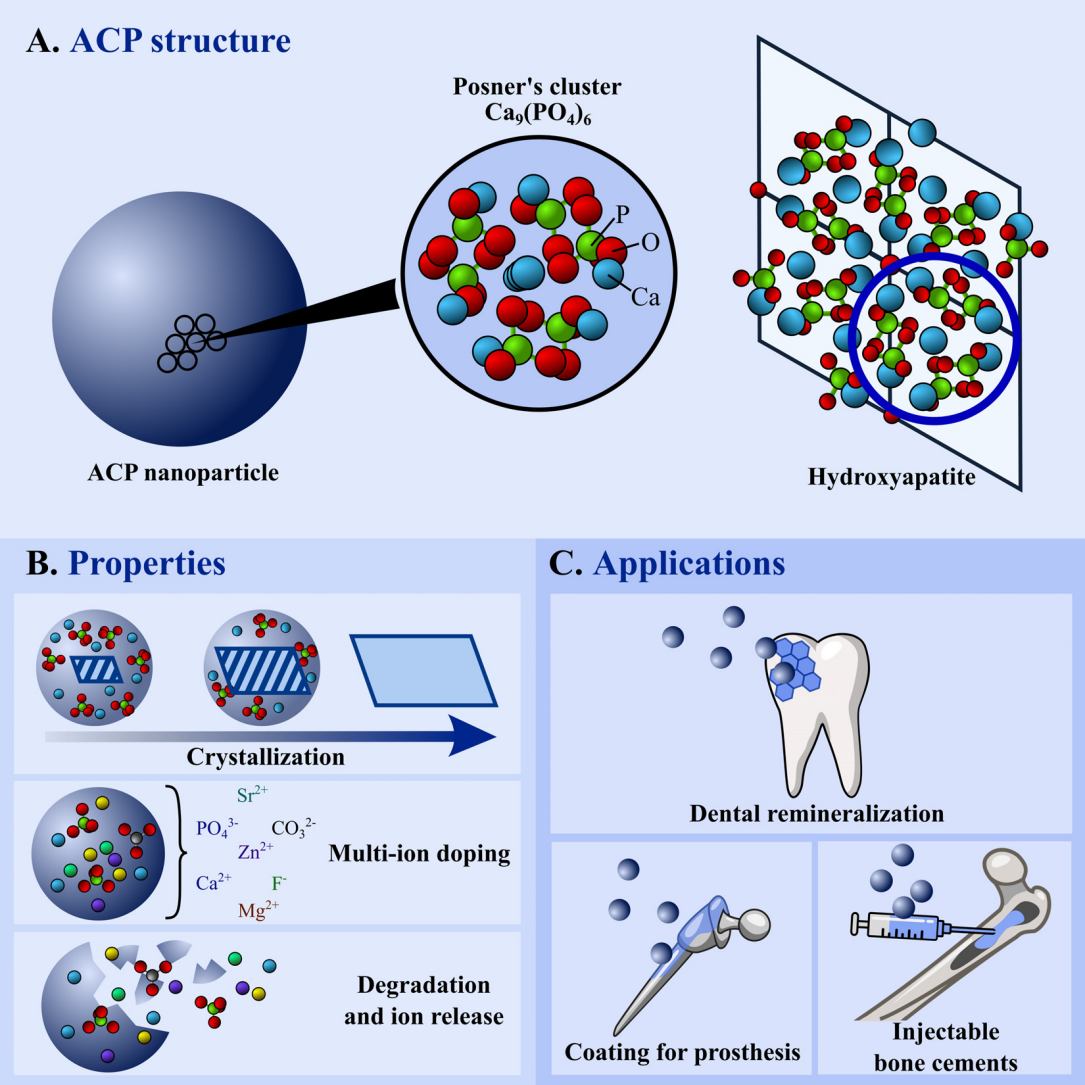
(A) Structure of ACP, Posner's cluster and the relationship with hydroxyapatite unit cell. (B) Properties and (C) Applications of ACP.

ACP can be represented by the general formula Ca_x_(PO_4_)_y_•nH_2_O. The basic structural unit of ACP is the Posner's cluster (Ca_9_(PO_4_)_6_), which is surrounded by 3–5 water molecules that “glue” several clusters together (Fig. 1A). However, ACPs can have different stoichiometry and local arrangement of atoms and a rough classification can be done on the base of their Ca/P molar ratio. ACP possess unique properties respect to crystalline CaPs due to its metastable nature (Fig. 1B) and it has a much higher degradability than any other calcium phosphates. This means that as soon as ACP is in contact with an aqueous media it starts to release Ca^2+^, PO_4_^3−^, and eventually other ions or molecules in the local environment. The non-rigid structure of ACP can host several foreign ions in higher amounts and variety than in the lattice of crystalline CaPs. ACP spontaneously crystallizes in presence of water, forming CaPs that are closely similar to the biogenic ones, which usually are apatite nanocrystals.

ACP has found in the last years interesting applications in the biomedical field. Probably, the most successful use is as dental remineralization agent (Fig. 1C). In dentistry, the loss of mineral from enamel due to cariogenic bacteria or by intake of acids is a relevant issue and is the first step of carious lesions. Remineralizing agents can provide Ca^2+^ and PO_4_^3−^ ions to increase the ionic supersaturation in saliva and stimulate the regrowth of new apatite crystal onto enamel, or they can directly attach and restore enamel surface. ACP can perform both actions, as it attaches to demineralized enamel surface, crystallize into apatite, and release its constituent ions to induce remineralization [3]. The most used ACP product for this application is ACP stabilized by casein phosphopeptide (CPP-ACP). Being one of the first marketed ACP-based product for dental remineralization under the trademark Recaldent^TM^, several papers and systematic reviews on the clinical evidences of CCP-ACP were published, see for example the review of Pithon et al. [4]. Enamelon^TM^ was another dental product based on un-stabilized ACP, where calcium and phosphate salts were delivered separately from a dual chamber device. The mixing of calcium with phosphate ions resulted in the immediate precipitation of ACP. Despite interesting evidences were provided on the efficacy of Enamelon^TM^, the dual-chamber system was challenging so it disappeared from the marketplace and was substituted by Enamelon®, a follow-up version of the older Enamelon™ that contains a polymeric complex of calcium and a phosphate salt. Other alternative and promising ACP products for enamel and dentin remineralization, as citrate-stabilized ACP, were developed more recently [5]. ACP has found also other successful applications in the field of biomaterials such as coatings for metallic or polymeric bone prostheses to improve osteointegration. ACP can be deposited onto the implants by both high and low energy techniques, for example by plasma spray deposition or biomimetic precipitation. Usually, after deposition ACP is partially transformed in an apatite layer through a heat- or water-induced crystallization processes. *In vivo*, ACP favors a fast fixation of the prosthesis to bone tissue [6]. Another application is for self-setting injectable bone cements, which are moldable pastes that can fit the shape of defects, leading to hardened bioactive materials favoring bone healing. The function of ACP is to hydrolyze in the paste to form apatite crystals which may entangle together to form a hard structure [7]. In comparison to other CaPs, the high reactivity of ACP allows to formulate cements with faster setting times. ACP was also used as nanomaterial for drug delivery exploiting its ability to adsorb or encapsulate higher amounts of bioactive molecules in respect to crystalline CaPs, in particular nucleic acids and proteins [8].

## Limitations

However, ACP-based products have some limits. First, the knowledge on ACP structure and composition is still incomplete. Indeed, the lack of crystalline order makes it more complex to study, as some investigation techniques are not suitable for amorphous materials (i.e. X-ray diffraction), or can induce an undesired crystallization events (electron microscopy and other high-energy techniques, analyses in wet conditions, etc.), and the results are complex and of difficult interpretation. There are several indirect proofs that ACP has polyamorphism, meaning that under the umbrella term “ACP” several distinct amorphous phases exist with different chemical composition, local order, and physicochemical behavior [6], but a systematic identification of these phases has yet to be done. Obviously, different ACPs should have different properties and applications, and therefore there is the need to (i) understand in detail how to control the ACP polyamorphism, (ii) which amorphous phases exist, and which are their properties, and (iii) how the local order and microstructure are correlated to macroscopic properties as dissolution and recrystallization.

Moreover, other hurdles of ACP are the preparation and stability. Indeed, ACP can be synthesized through a (i) wet precipitation or sol-gel reaction in aqueous or alcoholic solution, (ii) by high-energy milling, or (iii) by quenching of a melt. Every method has some drawbacks, as the wet precipitation and sol-reaction require the removal of solvent and unreacted precursors and have a limited yield, while mechanical and thermal syntheses are expensive in terms of energy consumption. Furthermore, the metastability of ACP is also a disadvantage, as limits the shelf life of ACP-based products and requires moisture-free storage conditions. In this case, the use of stabilizing agents that prevent crystallization has been widely studied. Namely these agents were (i) organic molecules, which adsorb on the surface of ACP particles such as previously mentioned CPP or citrate ions, or (ii) foreign ions such as Mg^2+^ or Sr^2+^ that, having a different size from Ca^2+^, inhibit the formation of an ordered structure.

## Future perspectives

Even considering these limitations, we believe that the future perspectives of ACP are bright. For example, ACP can be considered as a multi-functional material through its capability to deliver several ions at the same time. As dental remineralizing agents it should be prepared to simultaneously provide the basic building blocks of apatite (Ca^2+^, PO_4_^3−^), the ions that increase acid resistance (i.e. F^−^, as fluorapatite is less soluble than apatite), antibacterial ions (Ag^+^, Zn^2+^, etc.), enamel micro-constituents (Sr^2+^, Mg^2+^, etc.), and so on. In addition, ACP can be used as precursor for alternative preparation routes to synthetize CaPs biomaterials. Indeed it was demonstrated that through heating ACP can crystallize into non-stoichiometric apatite, allowing to consolidate and sinter massive bioceramics that have good mechanical resistance and high bioactivity [9,10].

## Conclusions

In the last decades ACP passed from being overlooked to being one of the most interesting materials for the development of advanced biomaterials. Its unique properties among CaPs such as high degradability, capability to deliver a high quantity of foreign ions, and the possibility to crystallize in a biomimetic apatite phase, have been harnessed leading to the development of excellent products in the field of dentistry, orthopedic surgery, and nanomedicine. Several works are needed to better understand the ACP structure at atomic level and the mechanisms of transformation to crystalline phases as well as to develop more efficient production methods. However, we envisage that the ACP's relevance and diffusion will continue to grow, and more and more successful applications will be discovered.

## Declaration of Competing Interest

M.I. declares two patent filings related to some of the technologies presented in this article (WO2016012452 filed on 21/07/2015 and WO2020002517 filed on 27/06/2019). The authors declare no other conflicts of interest.
